# Brain Oscillatory Activity during Tactile Stimulation Correlates with Cortical Thickness of Intact Areas and Predicts Outcome in Post-Traumatic Comatose Patients

**DOI:** 10.3390/brainsci10100720

**Published:** 2020-10-12

**Authors:** Galina Portnova, Irina Girzhova, Daria Filatova, Vitaliy Podlepich, Alina Tetereva, Olga Martynova

**Affiliations:** 1 Human High Nervous Activity Laboratory, Institute of Higher Nervous Activity and Neurophysiology of the Russian Academy of Science, 5A Butlerova str., 117485 Moscow, Russia; alina.tao@mail.ru (A.T.); omartynova@ihna.ru (O.M.); 2 Faculty of Medicine, Lomonosov Moscow State University, 27 Lomonosovsky pr-t., 119991 Moscow, Russia; girzhova.irina-103@yandex.ru (I.G.); dariafilatova.msu@mail.ru (D.F.); 3 Federal State Autonomous Institution N. N. Burdenko National Medical Research Center of Neurosurgery of the Ministry of Health of the Russian Federation, 16 4-ya Tverskaya-Yamskaya str., 125047 Moscow, Russia; podlepichv@gmail.com

**Keywords:** coma, unconsciousness, EEG, brain morphometry, TBI, tactile stimulation

## Abstract

In this study, we have reported a correlation between structural brain changes and electroencephalography (EEG) in response to tactile stimulation in ten comatose patients after severe traumatic brain injury (TBI). Structural morphometry showed a decrease in whole-brain cortical thickness, cortical gray matter volume, and subcortical structures in ten comatose patients compared to fifteen healthy controls. The observed decrease in gray matter volume indicated brain atrophy in coma patients induced by TBI. In resting-state EEG, the power of slow-wave activity was significantly higher (2–6 Hz), and the power of alpha and beta rhythms was lower in coma patients than in controls. During tactile stimulation, coma patients’ theta rhythm power significantly decreased compared to that in the resting state. This decrease was not observed in the control group and correlated positively with better coma outcome and the volume of whole-brain gray matter, the right putamen, and the insula. It correlated negatively with the volume of damaged brain tissue. During tactile stimulation, an increase in beta rhythm power correlated with the thickness of patients’ somatosensory cortex. Our results showed that slow-wave desynchronization, as a nonspecific response to tactile stimulation, may serve as a sensitive index of coma outcome and morphometric changes after brain injury.

## 1. Introduction

The relationship between brain damage and neurological mechanisms supporting recovery after severe brain injury has remained largely unknown [1]. The connection between behaviorally defined clinical entities and underlying brain damage has also been poorly investigated. However, magnetic resonance imaging (MRI) studies have demonstrated an association between the degree of tissue atrophy within the cortex, thalamus, basal ganglia, and basal forebrain and a patient’s quantitative clinical measures of behavioral responsiveness and arousal [2,3,4]. Peaks of maximal atrophy occurred in bilateral anterodorsal thalami, anterior dorsomedial caudate nuclei, and the anterior hippocampus [1]. After severe traumatic brain injury (TBI) without visible neuroradiological lesions, the volume of patients’ hippocampus, fornix, and other structures decreased [5]. According to Crick and Koch’s (2003) consciousness framework, brain networks, including sensory, semantic, and motor neural chains, are necessary for consciousness processes [6]. These neural coalitions could be described as a subset of cortical structures, including sensorimotor areas, the prefrontal cortex, the posteromedial cingulate cortex, and the parietal regions, as well as the relevant thalamocortical loops, which are capable of integrating the supervision, limited capacity, and re-entry properties of conscious sensory processing [7]. Impairment of the networks, including the frontoparietal and thalamocortical networks and encompassing the polymodal associative cortices, impaired consciousness [8,9,10,11,12,13].

However, data on the association between recovery after severe TBI and the level of brain damage are diverse. Recovery has been associated with the functional restoration of the frontoparietal network [10] and some of its thalamocortical connections [13]. Predicting patients’ likelihood of recovery from a coma relies on a comprehensive assessment of the ascending reticular activating system located in the posterosuperior part of the brainstem and structures encompassing the thalamus, basal forebrain, and frontoparietal association cortices [1,11]. Studies on traumatic coma patients with conventional MRI showed that lesions of the pons, midbrain, and basal ganglia were predictive of poor outcomes, especially when bilateral [14]. The length of the coma and memory loss were predicted by the reduction in callosal volume, which could be considered a marker of axonal loss [15]. A high frequency of deep supratentorial and stem lesions was correlated with a high incidence of coma and unfavorable outcomes, while cortical and subcortical white matter lesions were associated with good neurological status and prognosis. The lesions of the corpus callosum, subcortex, and stem induced a coma and increased the incidence of unfavorable outcomes [16]. According to Firsching et al. [17], establishing the exact location of primary and secondary brainstem lesions is crucial in predicting severe TBI outcomes.

Electroencephalography (EEG) has shown variable abnormal activity in comatose patients after TBI. Mecarelli et al. showed that background activity progressively decreased in amplitude until the eventual attenuation or suppression of cortical bioelectrical activity [18]. Spindle comas accompanied pontomesencephalic junction lesions when epileptiform activity and sleep spindles appeared along with the functional preservation of the remaining EEG patterns [19]. The beta coma pattern was most commonly induced by an acute brainstem lesion, while alpha coma patterns were detected in patients with pontomesencephalic hemorrhage or other acute encephalopathies [20]. Despite these findings, EEG patterns are not specific for a single etiology, and the general recovery prognosis depends on several factors, including the severity of the damage, type of injury, neurologic findings, and patients’ age [21]. However, individual EEG indices may aid the evaluation of outcome prognosis. For example, an alpha coma pattern and low voltage, slow, nonreactive patterns were generally considered biomarkers for poor outcomes [18,22], and spindle comas were associated with good outcomes [19]. Patients with bilateral independent periodic lateralized epileptiform discharges had a higher mortality rate than patients with the unilateral form [21].

The prediction of coma outcomes based only on MRI or EEG data is problematic. Previous studies have shown that a combined clinical and neuroimaging approach gives more accurate predictions [23]. Further, measures of reactivity to external responses are more informative than underlying EEG rhythms alone. Responses to auditory, noxious, tactile, and visual stimuli must be examined to assess changes in underlying patterns in steady-state EEG [24]. In particular, combining EEG in response to tactile stimulation and brain morphometry measures could be an effective method for predicting patients’ recovery from comas. The absence of a response to tactile stimulation may be a more useful predictor of poor outcomes than most clinical variables [12]. This assumption has been supported by data indicating the activating effect of tactile stimulation on human consciousness [25,26,27,28,29,30]. Additionally, tactile stimuli may cause a significant impact on the human emotional condition via C-tactile (CT) fibers, which respond vigorously to pleasant tactile experiences and support the affective–motivational dimension of touch [31]. Functional MRI (fMRI) studies have shown that preferential CT fiber stimulation activates the posterior insula cortex [32]. Several brain regions beyond the insula are also involved in the perception of tactile stimuli, including the right posterior superior temporal sulcus, medial prefrontal cortex, and cingulate cortex [33]. Studies in adult neuronopathy patients who lack Aβ fibers have shown that pure CT stimulation activates the insular cortex but not the somatosensory regions [28].

The stimulation of CT fibers affects the limbic system [31] and is a prospective approach in the rehabilitation of comatose patients. As a result, it should have a significant impact on patients’ recovery of consciousness. The evoked oscillatory response to tactile stimulation could also serve as a potential biomarker, predicting coma outcome. In the present study, we hypothesized that tactile stimulation could have a stimulating impact on unconscious patients after severe TBI affecting both Aβ and CT fibers, which have different cortical projections. We compared oscillatory brain activity during resting state and in response to tactile stimulation in healthy controls and TBI patients. Then, we investigated the correlation between EEG indices, brain morphometry data, and coma outcomes in TBI patients. Structural MRI supported the lack of damage to brain areas connected with tactile afferentation.

## 2. Methods

### 2.1. Participants

In total, 10 severe TBI patients (eight males and two females) aged 37.8 ± 29.8 years (M ± 2σ) participated in the study via inpatient observation at the N. N. Burdenko National Scientific and Practical Center for Neurosurgery from 2017 to 2018. The average length of total hospital stay after TBI (including time spent in the Intensive Care Unit) was 22.05 ± 57.58 days. The causes of severe TBI were as follows: car accident, fall from a height, and assault. Outcomes were as follows: transfer to another hospital, transfer to a rehabilitation center, recovery, and death (Table 1). The Glasgow Outcome Scale-Extended (GOSe) was used for objective outcome assessment. Exclusion criteria included a history of psychiatric or neurological disorders and the presence of seizures or epileptiform activity on EEG. All patients were observed and medicated according to guidelines for the management of severe TBI (4th edition) [34]. The depth and quality of sedation was assessed based on intracranial pressure parameters. Sedatives were stopped as soon as intracranial hypertension decreased.

The control group consisted of 15 healthy volunteers (nine males and six females) aged 29.6 ± 3.5 years old. Exclusion criteria included pregnancy, psychiatric or neurological disorders, and the use of any interfering medications or recreational drugs. Healthy participants reported having no current or past neurological or psychological disorders. Participants were informed about the experimental procedure, and the study was conducted in accordance with the Helsinki Declaration, while the study protocol was approved by the Ethics Departments of the Institute of Higher Nervous Activity and Neurophysiology of RAS and N. N. Burdenko National Medical Research Center of Neurosurgery. All healthy participants and patients’ legal representatives provided written informed consent.

### 2.2. Procedure

Patients were recruited for the EEG study within 12–48 h after MRI. Patients took part in the EEG study soon after MRI, and healthy controls participated in the EEG study within 1–2 weeks after MRI. In the neurological assessment of some patients, we used somatosensory evoked potentials (SSEPs) to exclude hand sensitivity impairment. Before the stimulation procedure, we recorded 5–7 min of resting-state EEG. Healthy controls were asked to close their eyes in resting state and under stimulation during the EEG recording to approximate experimental conditions to EEG registration in patients. Tactile stimulation was applied to the hairy part of participants’ left forearms. The types of stimuli and place of application were chosen for targeted stimulation of both αβ and CT fibers. Each stimulus was presented for 10 s in a randomized order with an interval of 2–10 s. Each stimulus was presented 10 times. A stimuli delivery sequence was composed in Presentation (Neurobehavioral Systems, Inc., CA, USA), which visualized stimuli type and presentation speed via an animation. An operator was trained to deliver stimulation according to the animation speed.

### 2.3. Stimuli

Four types of tactile stimulation were used, which were as follows:

(1) Slow stroking (2–3 cm/s) with a soft brush (60 mm, squirrel hair);
(2) Fast stroking (12–15 cm/s) with a soft brush (60 mm, squirrel hair);
(3) Slow stroking (2–3 cm/s) with a hard brush (60 mm, natural bristle);
(4) Fast stroking (12–15 cm/s) with a hard brush (60 mm, natural bristle).

The four selected types of tactile stimuli should stimulate different tactile receptors. The other reason to use different stimuli was the fast fatigue of tactile afferents (a phenomenon called “delayed acceleration” [29]). We selected four types of stimuli with the highest ratings by scales “Unpleasant–Pleasant” and “Slow–Fast” to reduce fatigue. Healthy participants reported that they perceived the first two types of stimulation as pleasant and the last two as unpleasant.

### 2.4. Subjective Assessment of Stimuli

Healthy controls reported on five aspects of the stimuli, including their painfulness, pleasantness, ticklishness, speed, and intensity.

### 2.5. EEG Registration

In this study, EEG was acquired using a 19-channel EEG amplifier, Encephalan, with a recording of polygraphic channels (Poly4, Medicom MTD, Taganrog, Russian Federation). The sampling rate was 250 Hz. The amplifier bandpass filter was nominally set to 0.05–70 Hz. Silver chloride (AgCl) electrodes (Fp1, Fp2, F7, F3, Fz, F4, F8, T3, C3, Cz, C4, T4, T5, P3, Pz, P4, T6, O1, and O2) were placed according to the International 10–20 system. Electrodes placed on the left and right mastoids served as joint references under a unipolar montage. The vertical electrooculogram (EOG) was recorded with AgCl cup electrodes placed 1 cm above and below the left eye, and the horizontal EOG was acquired by electrodes placed 1 cm lateral from the outer canthi of both eyes. Electrode impedances were kept below 10 kΩ. EEG fragments did not contain any epileptiform activity (which was exclusion criteria for patients). According to the American Clinical Neurophysiology Society (ACNS) nomenclature, all participants (both patients and controls) had symmetrical background EEG. All participants in the control group showed reactivity, posterior dominant alpha rhythm with eyes closed, clear anterior–posterior gradients, and normal voltage. Patients had normal or low voltage background EEG, absent or unclear reactivity, and reverse or no anterior–posterior gradients. Most patients had predominant background EEG with slow-wave frequency (2–5 Hz).

### 2.6. EEG Data Analysis

Fast Fourier transform was used to analyze power spectral density (PSD). The PSD was estimated for continuous artifact-free 200 ± 17.8 s epochs in resting-state EEG and 200 s segments merged from 10 s epochs of EEG corresponding to pleasant and unpleasant tactile stimulation. The resulting normalized spectra were integrated over intervals of unit width in the range of interest (2–3 Hz, 3–4 Hz, …, 19–20 Hz) for all conditions of stimulation and resting state.

### 2.7. MRI Acquisition

Control group MRI was performed using a 3T scanner (Magnetom Verio, Siemens, Erlangen Germany) at the National Research Center at the Kurchatov Institute in Moscow, Russia. Patient MRI was conducted after TBI by a 1.5 T scanner (Signa Horizon, General Electric, Boston, MA, USA) at the N. N. Burdenko National Medical Research Center of Neurosurgery in Moscow, Russia. For each participant, a sagittal high-resolution T1-weighted rapid gradient-echo (anatomical) image was acquired using a field of view 320 mm with a matrix size of 320 × 320a and a T1 MP-RAGE sequence as follows: TR 1470 ms, TE 1.76 ms, and FA 9°. In total, 176 slices with a slice thickness of 1 mm and a slice gap of 0.5 mm were reviewed.

### 2.8. Structural Morphometry

Brain morphometry was analyzed in both groups (Table 2). Each image was processed with a FreeSurfer standard “recon-all” pipeline with default parameters [35]. The pipeline included the following steps: segmentation of the subcortical white and gray matter volumetric structures (including the ventricles, amygdala, hippocampus, caudate, and putamen) [36,37]; intensity normalization [38]; tessellation of the gray matter and white matter boundary; automated topology correction [39,40]; and surface deformation following intensity gradients to place the gray/white and gray/cerebrospinal fluid borders at the optimal location, where the most significant shift in intensity defined the transition to the other tissue class [41,42,43]. When the cortical model was complete, deformable procedures were performed, including surface inflation [44], registration to a spherical atlas that utilized individual cortical folding patterns to match cortical geometry across subjects [45], parcellation of the cerebral cortex into units based on gyral and sulcal structures [46], and the creation of a variety of surface-based data, including maps of curvature and sulcal depth [43]. Cortical thickness and the voxel volume of subcortical structures were extracted using “aparcstats2table” and “asegstats2table” commands for each participant. Cortical parcellation was acquired according to the Desikan–Killiany Atlas [46].

### 2.9. Statistical Analysis

#### 2.9.1. Between and Within-Group Comparison

Brain morphometric values were compared between controls and patients using a nonparametric test. We used the Mann–Whitney U test to compare the whole grey matter volume, the volume of damaged brain tissue, the thickness of cortical anatomical structures, and the volume of subcortical anatomical structures parceled from anatomical atlases [38,39,46] in patients and healthy controls. To estimate between-subject and within-subject differences in PSD, we applied ANOVAs for repeated measures with a between-group design (2 GROUP × 5 CONDITIONS) followed by post-hoc Bonferroni comparison (*p* < 0.05). PSD values were averaged for all 19 electrodes for each 1 Hz width frequency interval from 2 to 20 Hz, separately for each condition (rest and four types of stimuli). The ANOVA was repeated for merged frequency PSD values, which confirmed a significant group effect for broader frequency bands.

#### 2.9.2. Within-Group Correlations

For the patient group, the significant obtained differences between rest and stimulation conditions in the average PSDs of theta (4–6 Hz), alpha (11–13 Hz), and beta (17–20 Hz) bands at each electrode were taken as x-values for a Spearman’s correlation analysis. The correlation analysis was applied with a cluster-based permutation procedure with 500 permutations and Bonferroni correction (*p* < 0.05). This tested for a possible association between EEG changes, outcome, and structural morphometric values. The outcome for each patient was evaluated by the attending physician based on clinical signs. For this correlation, data comprised values representing the scores of clinical scales, the whole grey matter, the damaged tissue, and cortical and subcortical anatomical structures. The threshold was at the 2.5th and the 97.5th quantiles. The samples were selected if the t-value was larger than the threshold of 0.025 and were clustered at a minimum of 1 electrode. Cluster-level statistics were calculated by taking the sum of the t values within every cluster. The values, which were outside the lower bound, obtained the same *p*-value (0.002).

#### 2.9.3. Comparison of PSD in Rest and Stimulation at the Individual Level

The individual differences in theta rhythm (4–6 Hz), alpha rhythm 11–13 Hz, and beta rhythm PSD between tactile stimulation (4*10 trials) and the resting state were calculated for each patient or subject of the control group separately using the Wilcoxon rank test with the Bonferroni correction (*p* < 0.0026). Each trial for each stimuli type (see Methods) was 10 s long; 40 artifact-free 10-s resting-state EEG fragments were used for the statistical comparison with 40 stimulation trials.

## 3. Results

### 3.1. Brain Atrophy in Coma Patients

**Figure 1:**
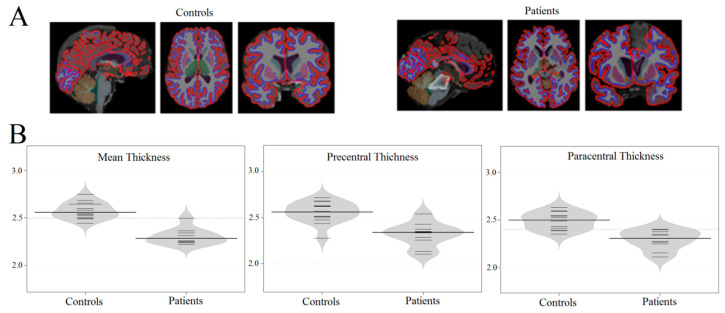
The morphometry results obtained by Freesurfer. **A**: examples of individual morphometry (for patients and healthy participants) showing the difference in the cortical thickness between comatose patients and the control group. **B**: Bean plots of cortical thickness for three measurements: mean cortical thickness, thickness of paracentral area, and precentral area (small black whiskers—individual values of cortical thickness).

Structural morphometry showed that trauma-affected brain areas did not overlap with areas associated with tactile perception (see Table 1). Whole-brain cortical thickness in comatose patients was lower than that of healthy participants (Table 1, Figure 1; (F(1, 23) = 15.241, *p* = 0.00338; MS = 10.7). The gray matter volume of the cortex and subcortical structures was also significantly lower in patients than in healthy controls as shown in Table 2 (F(1, 23) = 14,397, *p* = 0.00196; MS = 13.2). The main between-group differences in cortical thickness on the right side were located in the insula, anterior cingulate, and the right paracentral, precentral, supramarginal, and inferior parietal areas. Main between-group differences on the left side were in the insula, anterior and posterior cingulate, and the precentral, paracentral, supramarginal, middle frontal, parahippocampal, inferior, and superior parietal areas.

### 3.2. Altered Resting-State EEG in Comatose Patients

Compared to that of the control group, the power of slow-wave activity (between 2 and 6 Hz) was significantly higher (F(1, 23) = 13.600, *p* = 0.00122; MS = 14.2), and the power of alpha (8–12 Hz) and beta (16–20 Hz) rhythms was significantly lower (F(1, 23) = 21.967, *p* = 0.00010; MS = 16.9) in comatose patients during rest.

### 3.3. EEG Response Tactile Stimulation

**Figure 2:**
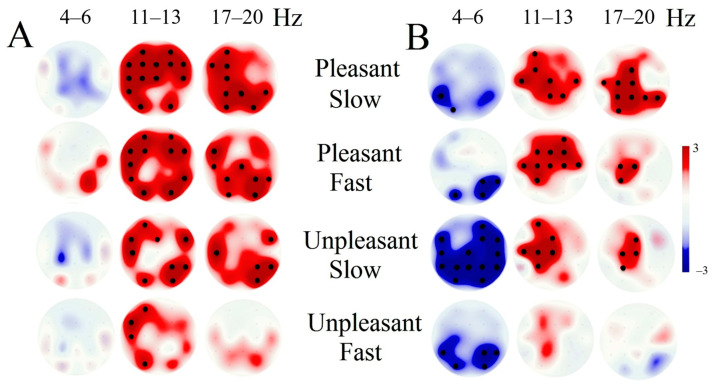
The difference in theta rhythm (4–6 Hz), alpha rhythm 11–13 Hz, and beta rhythm power spectral density (PSD) between tactile stimulation and the resting states for both groups of subjects. **A**: control group, **B**: patients in coma. The bold black dots indicate a significant difference after Bonferroni correction.

Tactile stimulation increased alpha (11–13 Hz) and beta (17–20 Hz) rhythm PSD in both groups (Figure 2).

During tactile stimulation, theta rhythm power in comatose patients decreased significantly compared to its value during resting-state EEG. This decrease was not observed in the control group (Figure 2; F(1, 23) = 11,694, *p* = 0.00234, MS = 11.7; Figure 2B). Greater decreases in theta rhythm power were correlated with better patient outcomes (*r* = 0.28, *p* = 0.02).

### 3.4. Individual EEG Response Tactile Stimulation

**Figure 3:**
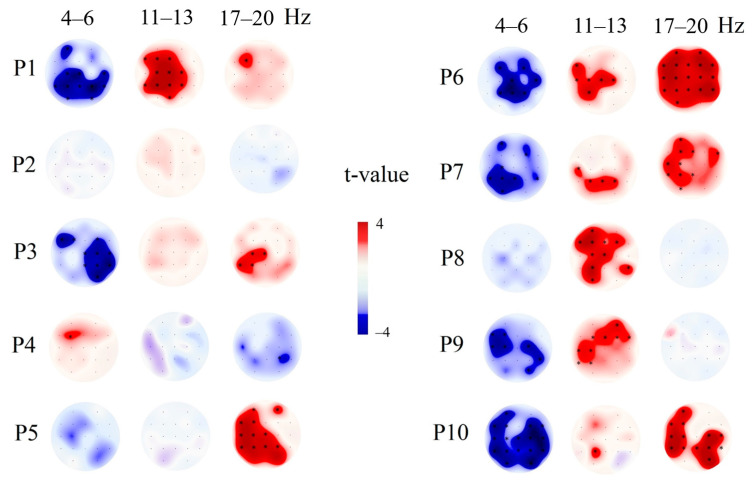
The individual differences in theta rhythm (4–6 Hz), alpha rhythm 11–13 Hz, and beta rhythm PSD between tactile stimulation (4*10 trials) and the resting state. P1-10—number of patient in accordance with Table 1. The individual differences between stimulus and rest were calculated using 4*10 trials of stimuli (each trial was 10 s long) and 40 10-s resting-state intervals. The differences were calculated for each patient separately; the Bonferroni correction was applied (*p* < 0.0026). The black stars indicate a significant difference after Bonferroni correction.

The individual differences of PSD between tactile stimulation and the resting state in the group of patients were depicted on Figure 3. According to individual data, the significant decrease of theta rhythm was not detected in patients with bad outcome (the lowest value of GOSe rate, see Table 1), in concordance with results of correlation analysis.

**Figure 4:**
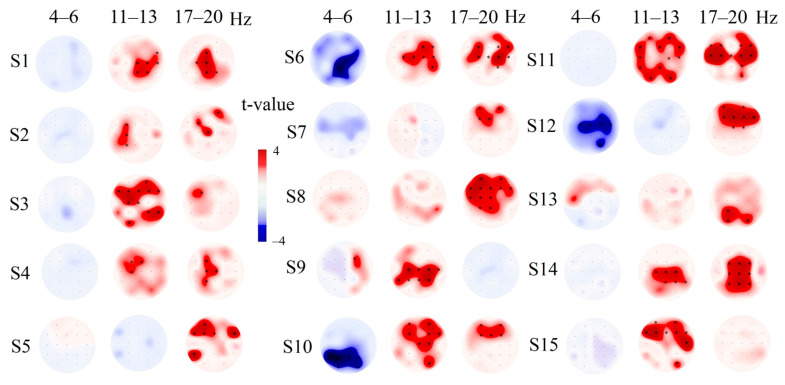
The individual differences in theta rhythm (4–6 Hz), alpha rhythm 11–13 Hz, and beta rhythm PSD between tactile stimulation (4*10 trials) and the resting state. S1-15—subjects of the control group. The individual differences between stimulus and rest were calculated using 4*10 trials of stimuli (each trial was 10 sec long) and 40 10-s resting-state intervals. The differences were calculated for each participant separately (*p* < 0.0026). The black stars indicate a significant difference after Bonferroni correction.

The individual differences of healthy participants are presented in Figure 4 and demonstrate the common trend of PSD changes during tactile stimulation described in within-group analysis. At the same time, individual variability was detected. In particular, three subjects from the control group showed a significant decrease of theta rhythm PSD compared to that found in the group of patients with good outcome.

### 3.5. Correlation of Tactile EEG Response with Morphometric Data

**Figure 5:**
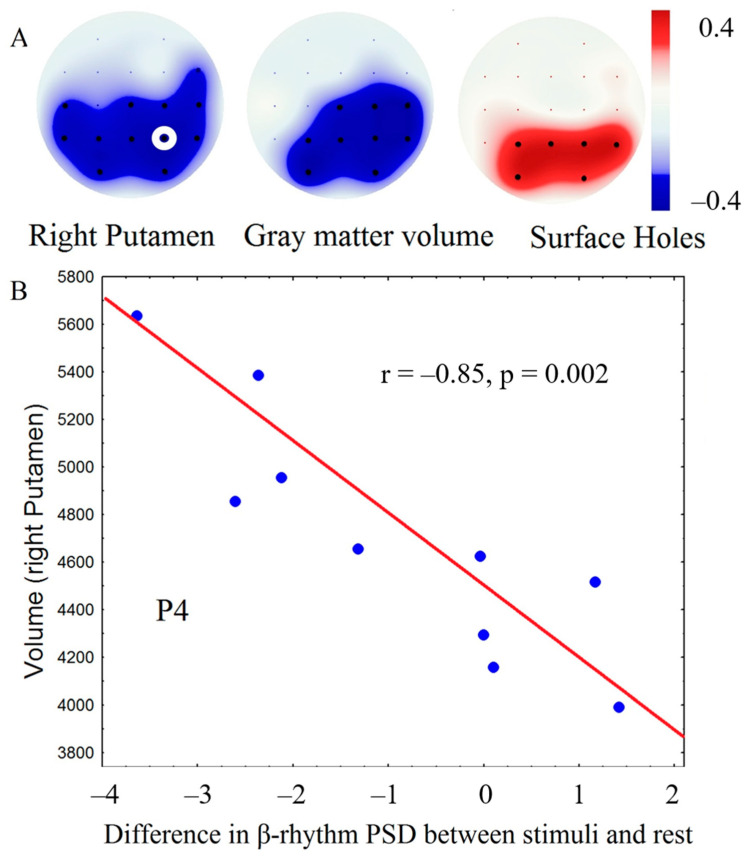
Results of clustering permutation’s correlation analysis between the volume of brain structures and average decrease of theta rhythm power during tactile stimulation compared to rest. **A**: maps of significant correlations (black dots—electrodes showing significant correlations after Bonferroni correction); **B**: scatterplot showing a relation between electroencephalography (EEG) spectral power at the electrode marked by a white circle and brain volume (x—difference between theta rhythm PSD during tactile stimulation compared to rest, y—volume in voxels), blue dots—individual values.

As shown in Figure 5, a decrease in theta rhythm (4–6 Hz) power in the occipital and parietal areas during tactile stimulation compared to rest was correlated with the volume of the right putamen, right insula, and whole brain gray matter and inversely correlated with the volume of damaged brain tissue (surface holes). Thus, the greater the decrease in theta rhythm power during tactile stimulation, the higher the volume of gray matter and the less brain damage was present.

**Figure 6:**
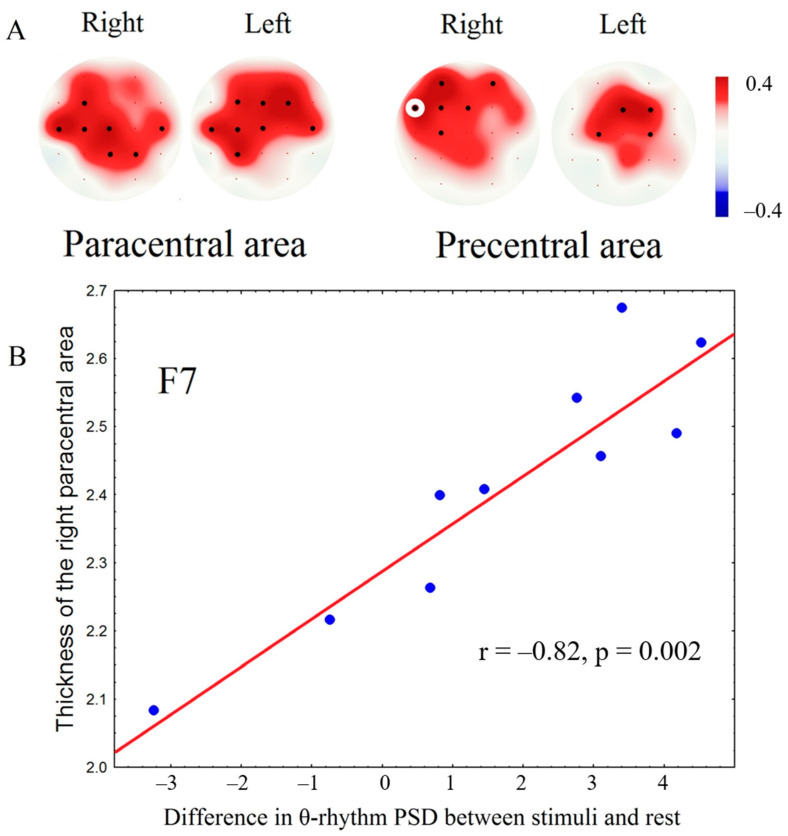
Results of correlation analysis between cortical thickness and averaged increase of beta rhythm power during tactile stimulation compared to the rest. **A**: maps of significant correlations (black dots—electrodes showing significant correlations after Bonferroni correction); **B**: scatterplot showing a relation between EEG spectral power at the electrode marked by a white circle and grey matter thickness (x—difference between beta rhythm PSD during tactile stimulation compared to rest, y—cortical thickness in mm), blue dots—individual values.

A correlation analysis between tactile EEG response and patient outcome showed that patients with better outcomes, such as recovery, had a more pronounced decrease in theta rhythm PSD during tactile stimulation (trend significance: *r* = −0.55, *p* = 0.01). The increase of beta rhythm (17–20 Hz) power in central areas was correlated with the thickness of paracentral (*r* = 0.82, *p* = 0.0005) and precentral (*r* = 0.80, *p* = 0.0007) areas bilaterally (Figure 6).

## 4. Discussion

Our findings indicated that the cortical thickness, the volume of the cortex, and the volume of subcortical structures were lower in comatose patients than in healthy participants. Our results were consistent with previous data, which showed that after severe brain injury, patients exhibited significant atrophy across all examined regions, including regions known to be involved in the regulation of electrocortical arousal, sleep–wake rhythms, and conscious behavior [13]. Extensive atrophy was detected in the globus pallidus, putamen, hippocampus, thalamus, caudate nucleus, brainstem, and basal forebrain [16].

Cortical atrophy could be associated with the traumatic reconstruction of the brain structure induced by injury and could impair tactile perception. We found that morphometry indices were significantly correlated with EEG responses during tactile stimulation in paracentral and precentral gyri only in comatose patients and could be critical for these processes. The greater the cortical thickness in central areas, the more pronounced the EEG response to tactile stimulation. Our findings indicated that an increase in beta rhythm PSD in response to tactile stimulation was correlated with the volume of variable cortical and subcortical thickness and the thickness of paracentral and precentral areas associated with sensory-motor networks but was mostly correlated with neural networks responsible for tactile perception [47,48]. The increase in beta rhythm power during stimulation correlated with the thickness of the paracentral and precentral areas. This response to tactile stimuli is well known and could be associated with stimuli processing activity [49]. The paracentral and postcentral areas are the primary somatosensory cortex locations, and each hemisphere receives tactile and proprioceptive perception from the opposite side of the body in the manner of the inverted homunculus. The secondary somatosensory cortex is in the vicinity of the lateral fissure and receives information from both sides of the body, and it has multiple connections with the premotor cortex, insular cortex, amygdala, and hippocampus [50].

The decrease in theta rhythm power during tactile stimulation in comatose patients correlated with the preservation of the brain from damage and was more prominent during unpleasant stimulation. Our results showed, at a trend level, that greater decreases in theta-band PSD were most pronounced in patients who went on to have better outcomes. In comatose patients, a significant response to tactile stimulation was registered at the theta rhythm frequency, while in healthy participants, this effect was absent. At the same time, analysis at the individual level demonstrated that 3 of 15 healthy participants (20 %) had a significant decrease of theta rhythm PSD during tactile stimulation, whereas only patients with the lowest (worst) outcome did not have a significant decrease of the theta-band PSD. We are discussing that despite a similar response, the decrease of theta rhythm PSD could have a different origin in comatose patients and healthy controls. First of all, the theta rhythm in coma patients is higher in the resting state than in healthy controls. This elevation of theta oscillations is induced by direct damage to the brainstem, diffuse axonal damage, or other etiologies, including cerebral hypoxia [51,52]. Further, continuous theta rhythm had been associated with poor outcomes [53]. In our study, the decrease of the theta-band PSD was found in patients with a better outcome, which could be a marker of their preserved sensitivity to tactile stimulation. Concerning healthy subjects, the decrease of theta rhythm PSD reflects memory and emotion processing [54]. In our study, the selective decrease of theta-band PSD could be associated in with individual cognitive or emotional reactivity to tactile stimulation.

Our findings indicated that tactile stimulation induced a response in comatose patients that was associated with brain regions related to consciousness. In particular, the global hallmark of impaired consciousness appears to be a multifaceted dysfunctional connectivity pattern with a within-network loss of connectivity in a widespread network of cortical and subcortical regions, including the insula and putamen [55]. In our study, the localization of brain regions correlated with comatose patients’ EEG response to tactile stimuli, and this response appeared to be carried out by at least two main functional awareness networks. These were the external network, or executive control network, subserved by variable cortical regions and the internal awareness network, or default mode network (DMN), which appears to be involved in self-related processes [56] and has been proposed as the locus of conscious awareness [57]. Previous studies also reported reduced connectivity in the DMN of comatose patients and those in a vegetative or minimally conscious state, which correlated with the level of consciousness.

## 5. Limitations

The overall implications of these results have been limited by the small sample size (10 patients and 15 healthy controls), which is associated with low statistical power and low reproducibility. In this regard, our study was supplemented by the individual event-related statistics of PSD differences in patients and controls. Further, the strength of Spearman’s correlation of EEG spectral power at several electrodes exceeded 0.8, which implied that 10 participants were enough to satisfy the power of analysis (α < 0.05, β < 0.2). The other significant limitation arises from the timing of EEG recording in the patient group after TBI. Patients’ clinical conditions meant that MRI was delayed for days or weeks after injury, as the clinical protocol permitted only computed tomography scanning. We aimed to compare EEG response to tactile stimulation with brain morphometry, so we recruited patients for the EEG study as soon as possible (about 12–48 h) after MRI acquisition. Accordingly, further longitude research is needed to compare EEG response to tactile stimulation and its relationship with cortical thickness depending on time passed after TBI. Additionally, it would be helpful to compare EEG response to tactile stimulation with EEG changes induced by other stimulation types in correlation with brain morphometric measures.

## 6. Conclusions

This study showed that EEG response to tactile stimulation correlated with brain damage after TBI and could predict coma outcome. We found that the cortical thickness and volume of the cortex and subcortical structures were lower in comatose patients than in healthy controls. The specific tactile response (shown by an increase in alpha and beta rhythms) in patients and healthy volunteers correlated with the thickness of the somatosensory cortex. The nonspecific response to tactile stimulation (shown by a decrease in spectral power in the theta range) was associated with the preservation of cortical and subcortical volume in brain areas known to be part of awareness supporting networks. Patients with good outcomes, such as recovery of consciousness, had a more pronounced decrease in theta rhythm PSD during tactile stimulation. Our findings suggest that specific and nonspecific oscillatory responses to tactile stimulation can indicate the severity of brain damage after TBI and serve as biomarkers for comatose patients’ rehabilitation.

## Figures and Tables

**Table 1:** Clinical information about patients.

Patient.	Cause of Trauma	Age	Time from Accident to EEG Recording	Coma Glasgow Scale	MRI Results (Regions of Brain Contusions)	Outcome (GOSe Rate)
1	car accident	18	7 days	5	right frontal and the corpus callosum	6
2	car accident	41	28 days	7	right temporal–frontal region left temporal–frontal region, right hemisphere of the cerebellum	3
3	car accident	43	12 days	7	front temporal lobes of the right hemisphere left temporal and occipital lobes	5
4	car accident	26	2 months 2 days	6	left frontal and right occipital lobe	3
5	Beating	32	1 month 18 days	5	brain stem left basal temporal region	3
6	fall from a height	51	7 days	6	left frontal and temporal regions, diffuse axonal damage	5
7	car accident	34	2 months 25 days	7	diffuse axonal damage	6
8	car accident	34	4 days	5	the corpus callosum, diffuse axonal damage	3
9	car accident	38	8 days	6	brain stem	5
10	car accident	26	4 days	5	the corpus callosum, diffuse axonal damage	7

**Table 2:** Cortical thickness in areas associated with tactile stimulation and level of consciousness (according to literature data) and their cross-group differences.

	Area	Thickness (mm) in Control Group (mean ± std)	Thickness (mm) in Comatose Patients (mean ± std)	Z (Mann–Whitney U Test)	*p*-Value
Right	Anterior cingulate	2.7 ± 0.3	2.5 ± 0.2	1.36	0.17
	Posterior cingulate	2.5 ± 0.2	2.5 ± 0.2	1.16	0.24
	Middle-frontal	2.6 ± 0.1	2.6 ± 0.2	0.78	0.44
	Parahippocampal	2.8 ± 0.3	2.8 ± 0.3	−1.14	0.26
	Inferior-parietal	2.5 ± 0.1	2.4 ± 0.1	1.83	0.07
	Superior parietal	2.2 ± 0.1	2.2 ± 0.1	−0.08	0.93
	Paracentral	2.5 ± 0.1	2.3 ± 0.1	2.22	0.03
	Postcentral	2.0 ± 0.1	2.0 ± 0.1	1.00	0.32
	Precentral	2.6 ± 0.1	2.3 ± 0.2	3.00	0.00
	Precuneus	2.4 ± 0.1	2.4 ± 0.1	−1.55	0.12
	Supramarginal	2.6 ± 0.1	2.4 ± 0.2	2.77	0.01
	Insula	3.1 ± 0.1	2.9 ± 0.2	3.16	0.00
	Mean Thickness	2.6 ± 0.1	2.1 ± 0.1	3.54	0.00
Left	Anterior cingulate	2.8 ± 0.2	2.7 ± 0.4	1.11	0.27
	Posterior cingulate	2.6 ± 0.1	2.4 ± 0.2	1.94	0.05
	Middle-frontal	2.5 ± 0.1	2.4 ± 0.2	0.89	0.37
	Parahippocampal	2.8 ± 0.3	2.9 ± 0.2	−0.83	0.41
	Inferior–parietal	2.5 ± 0.1	2.4 ± 0.1	1.22	0.22
	Superior–parietal	2.2 ± 0.1	2.1 ± 0.1	1.58	0.11
	Paracentral	2.4 ± 0.2	2.3 ± 0.1	1.55	0.12
	Postcentral	2.0 ± 0.1	2.0 ± 0.1	1.05	0.29
	Precentral	2.6 ± 0.1	2.3 ± 0.2	3.36	0.00
	Precuneus	2.4 ± 0.1	2.4 ± 0.1	0.64	0.52
	Supramarginal	2.6 ± 0.1	2.4 ± 0.2	2.58	0.01
	Insula	3.1 ± 0.1	2.8 ± 0.2	2.55	0.01
	Mean Thickness	2.6 ± 0.1	2.4 ± 0.1	3.05	0.00
